# Nutritional status at diagnosis and follow-up and its impact on short-term clinical outcome in children with cancer: a real-world report from India

**DOI:** 10.3332/ecancer.2026.2137

**Published:** 2026-06-02

**Authors:** Rajul M Gala, Maya Prasad, Shyam Srinivasan, Venkata Rama Mohan Gollamudi, Chetan Dhamne, Nirmalya Roy Moulik, Badira Parambil, Akanksha Chichra, Gaurav Narula, Girish Chinnaswamy

**Affiliations:** 1 Division of Paediatric Oncology, Tata Memorial Centre, Parel, Mumbai 400012, India; 2 Homi Bhabha National Institute (HBNI), Anushakti Nagar, Mumbai 400094, India; a https://orcid.org/0000-0003-0127-7987

**Keywords:** nutritional status, survival outcome, low-middle income countries, children, cancer

## Abstract

Nutritional status (NS) in children with cancer may impact a wide range of outcomes, and remediation of under and over-nutrition may potentially improve outcomes. Children with cancer at our centre were classified as undernourished (UN) body mass index (BMI Z-score ≤−2 standard deviations (SD) World Health Organisation (WHO) or mid-upper-arm circumference (MUAC) <12.5 cm for <5 years (WHO) or <25th percentile for >5 years (Frisancho)), well-nourished (WN) (BMI Z-score −1 SD to +1 SD or MUAC 12.5–13.5 cm for <5 years or 25–50th percentile for >5 years) and over-nourished (ON) (BMI Z-score ≥+1 SD or MUAC ≥75th percentile for >5 years). NS was assessed at three time-points: diagnosis, 3- and 6-month follow-up. Trends in NS and impact on outcomes (event-free survival (EFS) and overall survival) were analysed. At diagnosis (n = 2,086) and 6-month follow-up (n = 1,245), 30.9% and 41.7% were WN, 65.3% and 52.2% UN and 3.8% and 6.1% ON. During the course of treatment, 45.5% gained weight and 11.6% lost weight. The highest prevalence of undernutrition at diagnosis was seen in acute myeloid leukaemia (73.3%), lymphoma (68.8%) and bone tumours (68.7%). Two-year EFS was 71.3% ± 1.82% in WN children, 71.3% ± 5.19% in ON children and 68.4% ± 1.28% in children UN at diagnosis (p = 0.004). Children who gained weight between diagnosis and follow-up had a 2-year EFS of 82.8% ± 1.60% versus 76% ± 1.70% in those without weight gain (p = 0.022). Undernutrition at diagnosis as well as during treatment leads to increased relapse as well as mortality. Weight gain and improved NS appear to impact clinical outcomes, highlighting the need for targeted nutritional interventions in children with cancer.

## Introduction

The majority of children diagnosed with cancer across the world belong to low-income and low-middle income countries (LMIC) [1]. Undernutrition remains a critical global health challenge in these regions, responsible for approximately 45% of deaths in children under 5 years, which often results in patients being already malnourished at the time of their cancer diagnosis [2]. However, the outcomes of cancer treatment continue to be low in these settings, with toxic deaths and treatment abandonment being major causes of treatment failure [1]. Undernutrition is a key prognostic variable contributing to increased treatment-related toxicities (TRT) such as infections as well as poor tolerance to treatment, treatment delays, abandonment of therapy and poor quality of life all of which lead to inferior outcomes in children with cancer [3–6].

Certain tumour types (metastatic solid tumours, acute myeloid leukaemia (AML), high-risk and relapsed acute lymphoblastic leukaemia (ALL), brain tumours and nasopharyngeal carcinomas) and treatment exposures (high dose chemotherapy, hematopoietic stem cell transplantation, intensive phases of chemotherapy, radiation involving the brain and abdominal surgery) and demographic factors (low socioeconomic status and limited access to appropriate supplementation) are known to have a high risk of undernutrition [6]. Weight loss is common both at disease diagnosis and during cancer treatment, has multifactorial causes and necessitates appropriate and timely nutritional assessment and management [3–6]. There is emerging evidence that remediation of under and over nutrition may improve outcomes [7–9]. Employing an effective strategy for nutritional assessment and management is challenging, but has been shown to result in superior nutritional outcomes [10, 11].

Previous reports from our centre documented a high incidence of undernutrition in newly diagnosed children with cancer and at follow-up, which remains a major concern [10, 12]. In this paper, we analysed the trajectories in nutritional status (NS) at various time-points during treatment. We also analysed the impact of the NS on event-free (EFS) and overall-survival (OS)in a large cohort of children newly diagnosed with cancer.

## Materials and methods

This is a retrospective cohort study, which included patients (0–15 years) registered in the paediatric oncology unit between 1 January 2018 and 31 December 2019. All diagnoses were eligible. Children without reliable data on NS and clinical follow-up were excluded.

### Nutritional assessment

All children underwent nutritional assessment at diagnosis and at regular intervals throughout treatment, as per departmental protocol [10]. Parameters measured and documented were weight, height/length and mid-upper-arm circumference (MUAC). The *Z*-scores for weight-for-age, height-for-age, weight-for-height or body mass index (BMI) for age were computed for classification of NS.

Children aged ≤5 years were defined to have severe acute malnutrition (SAM) if MUAC was <11.5 cm or weight-for-height was <−3 standard deviations (SD), and moderate acute malnutrition (MAM) if MUAC was between 11.5 and 12.5 cm or weight-for-height was −3 SD to −2 SD. They were termed overweight (OW) if weight-for-height was between 1 SD and 2 SD and obese if weight-for-height was >2 SD. Children aged >5 years were classified as SAM if BMI-for-age was <−3 SD or MUAC for age was <5th percentile, and MAM if BMI-for-age was between −3 SD and −2 SD or MUAC 5–10th percentile, OW if BMI-for-age was between 1 SD and 2 SD or MUAC for age was >50th percentile, and obese if BMI-for-age was >2 SD. These definitions were as per World Health Organisation (WHO) classification for weight-for-height, BMI-for-age and MUAC in <5 years; and Frisancho classification for MUAC in >5 years [13, 14]. The remaining patients were grouped as well nourished (WN) (Supplementary Table 1).

For purposes of this manuscript, children who were SAM and MAM were termed in combination as undernourished (UN) and children who were OW and obese were termed over-nourished (ON).

### Nutritional intervention and follow-up

A detailed description of the nutritional management and intervention in our unit has been published previously [11]. Children with SAM and MAM were follow-up weekly and 2-weekly, respectively, until optimum weight was achieved and monthly thereafter [15]. Those who were WN were monitored monthly if intake was good and no weight loss was anticipated. Those who were OW and obese were followed-up 3- and 2-weekly, respectively [10].

### Clinical follow-up

The clinical end-points documented for this study were treatment abandonment, disease progression, recurrence, toxic death and death due to other cause.

### Protocols for treatment of specific cancers

Children with specific cancer diagnoses were treated according to standard, predominantly institutional treatment protocols detailed in Supplementary Table 4.

### Data collection

Data were collected from the division of Paediatric Oncology, Tata Memorial Centre, Mumbai. For the purpose of this study, diagnoses were grouped as ALL, AML, lymphoma, brain tumours, head and neck tumours, bone tumours, abdominal solid tumours (non-lymphoma) and other solid tumours.

The clinical and demographic data (age, sex, diagnosis, treatment details and follow-up) were collected from the department database and nutrition data (height, weight, BMI, MUAC and biochemical parameters at diagnosis and follow-up) were extracted from the department nutritional database [16]. Socio-economic status was also considered, with most children in this cohort belonging to a poor socio-economic strata, which is one of the important determinants of NS as highlighted in the introduction. Data on treatment-related complications were not included in the analysis; however, most of the worsening of NS during treatment may be attributable to treatment-related complications. Both prospective databases are maintained real-time by data managers and nutritionists, respectively. Weight gain was defined as >10% gain in weight and weight loss was defined as >10% loss in weight from diagnosis to 3-month follow-up. Data on NS were taken at diagnosis, 3 months from diagnosis and 6 months from diagnosis (where available). Improvement in NS was defined as a change from either SAM to MAM, from MAM to WN status or from OW/obesity to WN status. Worsening of NS was defined as a change from WN status to MAM or OW status or obesity or from MAM to SAM.

The Institutional Ethics committee approved this retrospective analysis and waiver of consent was obtained.

### Statistical analysis

All demographic data were summarised as descriptive statistics, with continuous data as median and range or interquartile range (IQR). EFS was defined as the time interval between the date of diagnosis and the date of event (relapse, second malignancy, treatment abandonment, death) or last follow-up whichever occurred earlier. OS was defined as the time interval between date of diagnosis and date of death due to any cause or the date of the last follow-up. Patient follow-up was updated until January 2023. Survival analysis was done by Log-Rank test and presented as Kaplan-Meier curves. Univariate analysis was performed by Cox proportional hazard model. Analysis was performed using IBM SPSS™ Statistics for Windows software, version 25.0. The following variables were analyzed for their impact on EFS and OS: NS at diagnosis and follow-up, weight gain, weight loss and improvement in NS. Univariate and multivariate analyses were performed by Cox proportional hazard model. For the purpose of multivariate analysis, variables with a *p*-value less than 0.2 on univariate analysis were included.

## Results

### Demographics

**Figure 1.**
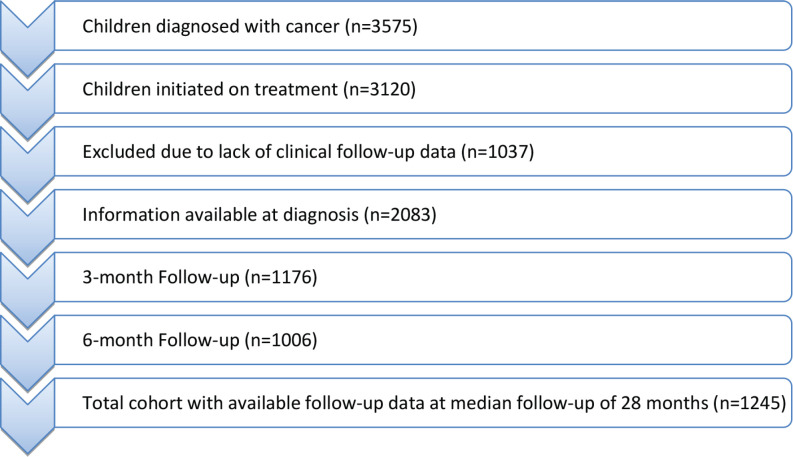
Study enrollment and follow-up flowchart.

Over the study period of 2 years, 3,575 children were diagnosed with cancer at our centre, and 3,120 children were initiated on treatment (Figure 1). Information on NS and clinical outcome was available in 2,083 children at diagnosis and 1,245 children at follow-up (Figure 1). The cohort was predominantly male, with a median age of 6.5 years (IQR 3.3–11 years). The most frequent diagnosis was ALL (40%), followed by abdominal solid tumours (12.7%), lymphomas (12%) and bone tumours (11%) (Table 1).

At a median follow-up of 28 months, 65 (3.1%) either abandoned treatment or were lost to follow-up, 1,322 (63.5%) are alive in first remission, 545 (26.2%) relapsed or progressed on treatment and 151 (7.2%) expired due to TRT. Of those who relapsed or progressed on treatment 106 (19.4%) are alive in second remission (Table 1).

### Trajectory of NS

**Figure 2.**
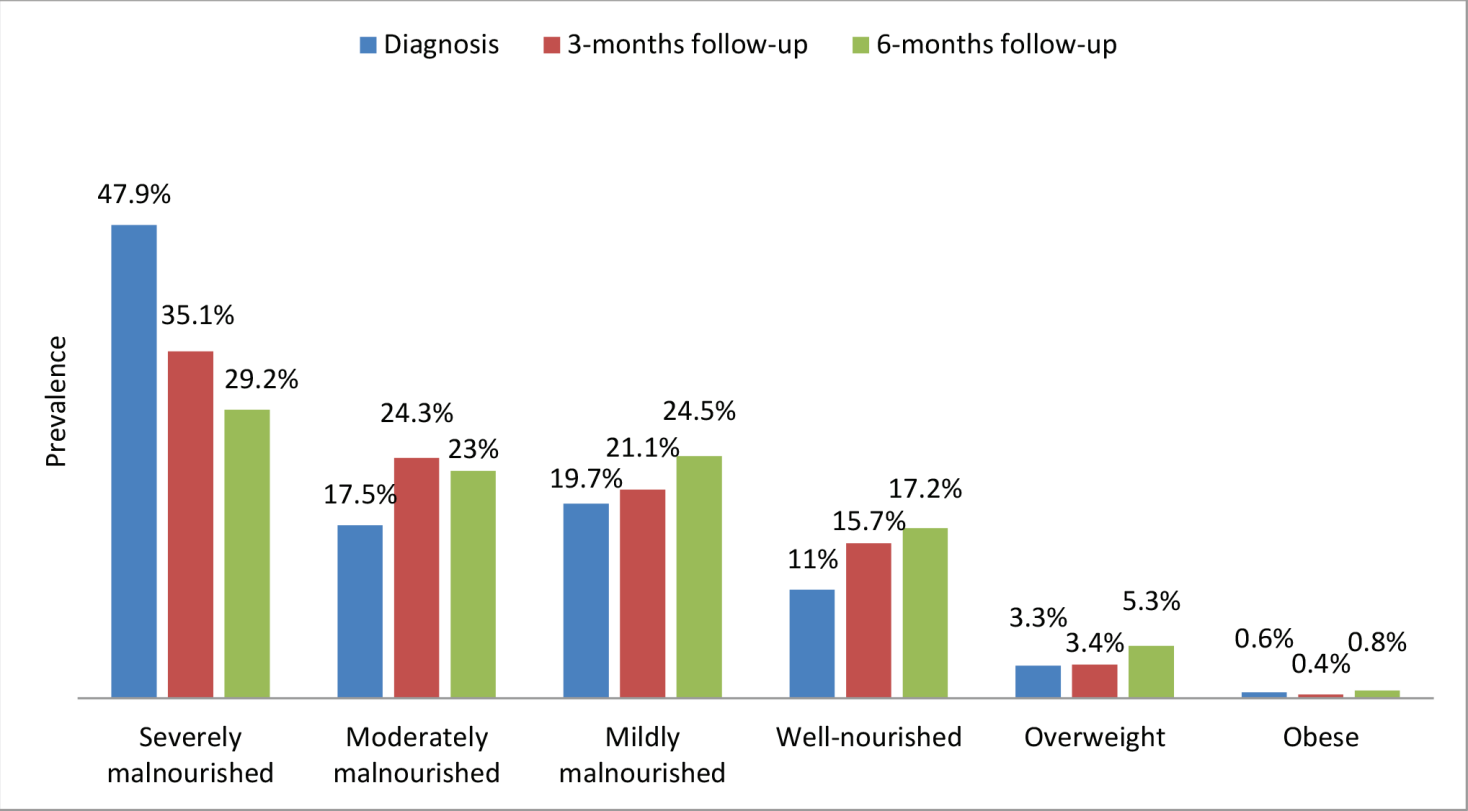
Trends in NS at diagnosis and follow-up.

At diagnosis (*n* = 2,083), 233 (11%) were WN, 410 (19.7%) were mildly malnourished, 363 (17.5%) were moderately malnourished, 997 (47.9%) were severely malnourished, 68 (3.3%) were OW and 12 (0.6%) were obese (Figure 2).

During the course of treatment (*n* = 1,245), 566 (45.5%) gained >10% weight, 145 (11.6%) lost >10% weight and 534 (42.9%) either showed <10% change or no change in weight. Improvement in NS was seen in 254 (20.4%) children, while the rest either worsened or had no change in their NS (Table 2).

**Supplementary Figure S1.**
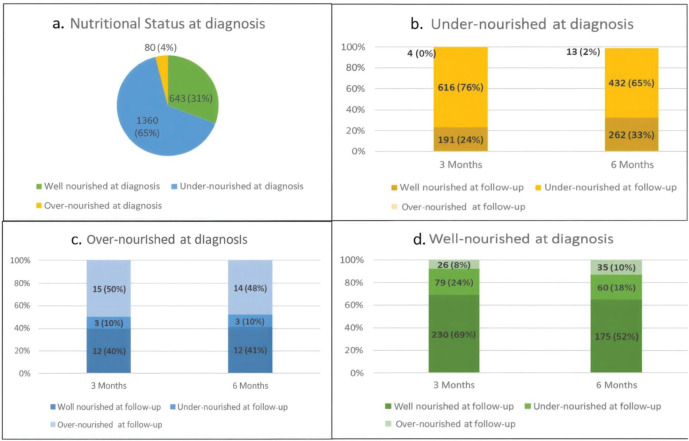
NS at diagnosis (*n* = 2,083) and follow-up (n = 1,245) (a): NS of the entire cohort at diagnosis (b): NS at follow-up of those who were UN at diagnosis (c): NS at follow-up of those who were ON at diagnosis (d): NS at follow-up of those who were WN at diagnosis.

Of the 2,083 children, data were available in 1,176 at 3-month follow-up and 1,006 at 6-month follow-up. Of 335 children who were WN at diagnosis, 230 (68.7%) remained WN at 3 months, while 79 (23.6%) children worsened to become UN and 26 (7.8%) children became ON. Out of 811 children who were UN at diagnosis, 191 (23.6%) became WN while 4 (0.5%) became ON at 3-month follow-up. Out of 30 children who were found to be ON at diagnosis, 12 (40%) became WN while 3 (10%) became UN at 3 months. Most children (65%) who were WN at diagnosis (*n* = 270) continued to remain WN at 6-month follow-up, 60 (22.2%) children became UN, while 35 (13%) children were ON. Of the 707 children who were UN at diagnosis, 232 (32.8%) became WN, while 13 (1.8%) became ON. Out of 29 children who were ON at diagnosis, 12 (41.4%) became WN while 3 (10.3%) became UN at 6 months follow-up. (Supplementary Figure S1)

### Changes in NS by diagnosis

**Supplementary Figure S2.**
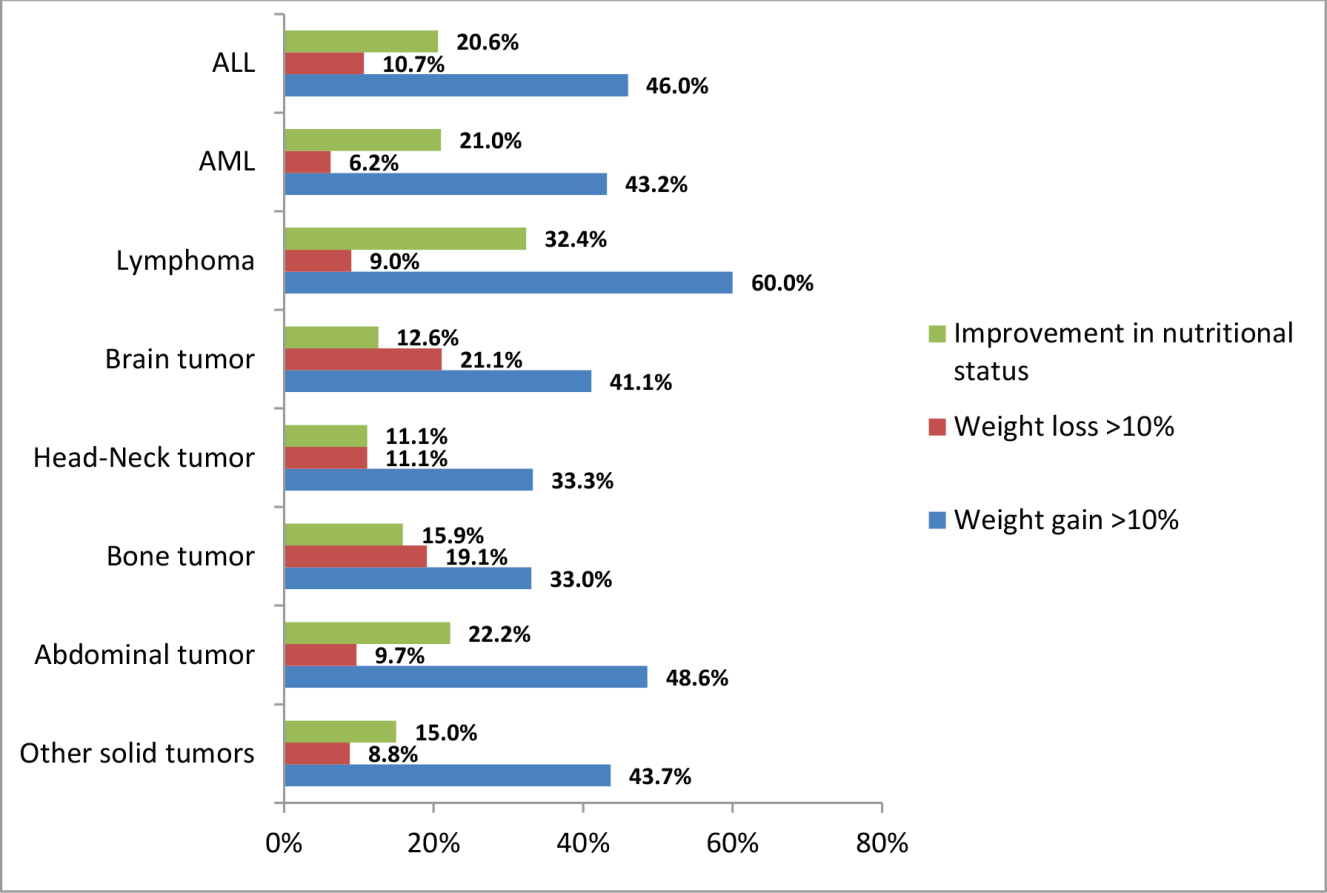
Changes in weight trend by diagnosis.

The prevalence of undernutrition at diagnosis was highest in children with AML (96, 73.3%) and lymphoma (172, 68.8%), at 3-month follow-up in children with brain tumour (57, 67.1%) and bone tumour (82, 65.1%) and at 6-month follow-up in children with head-neck tumour (20, 69%) and bone tumour (75, 68.8%) (Table 3). Weight loss was highest in children with brain tumour (21.1%) and bone tumour (19.1%) and weight gain was highest in children with lymphoma (60%) and abdominal tumours (48.6%) (Supplementary Figure S2).

### Associations between NS and clinical outcome

**Supplementary Figure S3.**
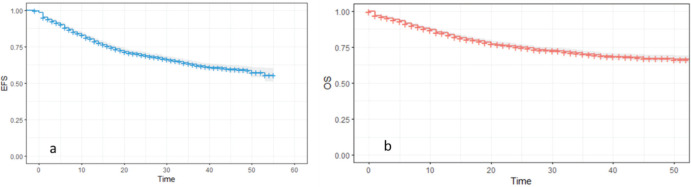
(a): EFS and (b): OS of the entire cohort.

Outcomes: For the entire cohort, EFS and OS at 24 months were 69.7% ± 1.00% and 75.8% ± 1.00% (Supplementary Figure S3).

**Figure 3.**
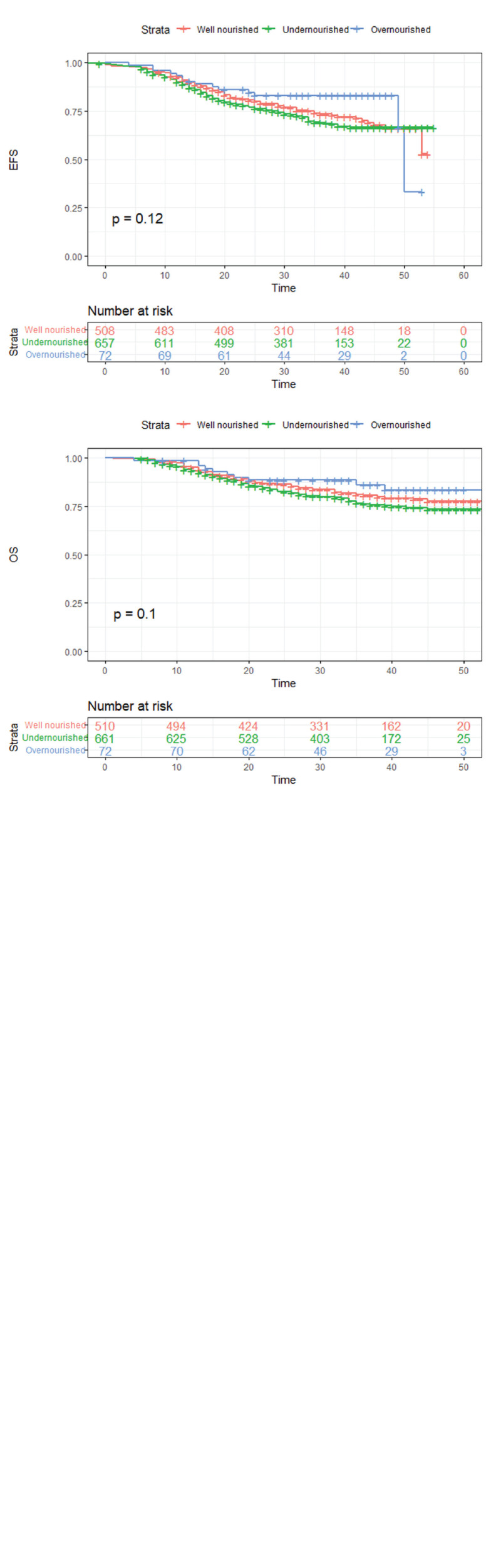
EFS and OS by NS at diagnosis and NS at follow-up: (a): EFS by NS at diagnosis (b): OS by NS at diagnosis (c): EFS by NS at diagnosis NS at follow-up (d): OS by NS at diagnosis NS at follow-up.

NS and outcomes: The 24-month EFS in children who were WN (*n* = 643) at diagnosis was 71.3% ± 1.82% and ON (*n* = 80) at diagnosis was 71.3% ± 5.19% versus 68.4% ± 1.28% in those who were UN (*n* = 1,360) at diagnosis (*p* = 0.004). The 24-month OS of those who were WN at diagnosis was 77.5% ± 1.70% and ON at diagnosis was 75.5% ± 5.05% versus 74% ± 1.22% in those who were UN at diagnosis (*p* = 0.085) (Figure 3, Supplementary Tables 2 and 3).

Figure 3 illustrates the impact of NS at follow-up on outcomes.

**Supplementary Figure S4.**
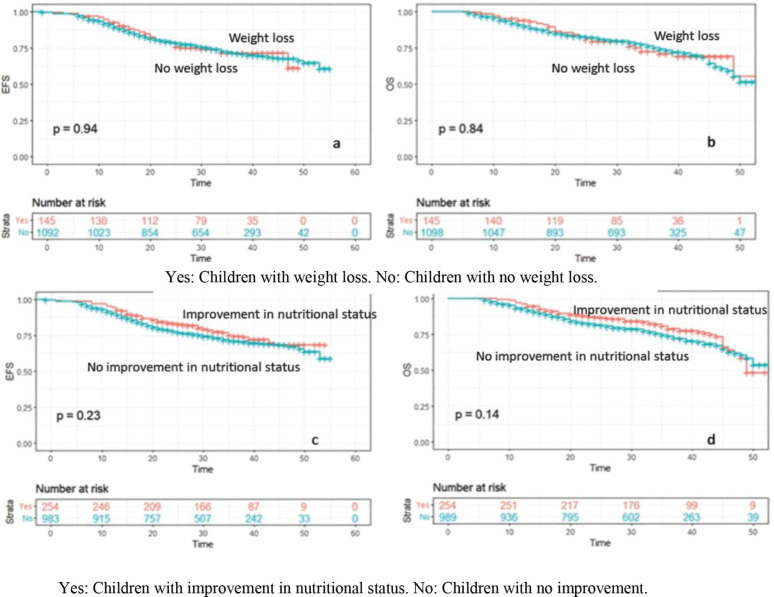
FS and OS by weight loss and improvement in NS. There was no significant difference in (a): EFS and (b): OS of those with weight loss compared those without weight loss or in (c): EFS and (d): OS of those with improvement in NS compared to those without improvement.

**Figure 4.**
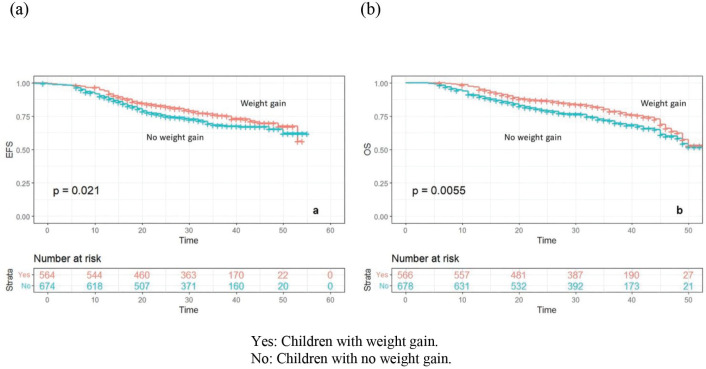
EFS and OS by weight gain from diagnosis to follow-up: (a): EFS by weight gain from diagnosis to follow-up (b): OS by weight gain from diagnosis to follow-up. Both EFS and OS at 12 and 24 months were significantly higher in children who gained weight compared to those who did not.

Children who gained weight between diagnosis and follow-up had a 24-month EFS of 82.8% ± 1.60% versus 76% ± 1.70% in those who did not gain weight (*p* = 0.021) (Figure 4). Those who lost weight between diagnosis and follow-up had a 24-month EFS of 78.2% ± 3.50% versus 79.2% ± 1.20% in children who did not lose weight (*p* = 0.98). Children who showed improvement in their NS between diagnosis and follow-up had a 24-month EFS of 83.1% ± 2.40% versus 78.1% ± 1.30% in those without improved NS (*p* = 0.282). Children whose NS improved between diagnosis and follow-up had a 24-month OS of 87.9% ± 2.10% versus 83.7% ± 1.20% in those whose NS was stable or worsened (*p* = 0.085) (Supplementary Figure S4). Children with ALL who experienced weight gain showed a 24-month OS of 88.5% ± 2.10%, compared to 82.1% ± 2.30% in those without weight gain (*p* = 0.045). Children with other solid tumours who gained weight achieved 91.3% ± 4.80% OS compared to 81.1% ± 6.00% in those without weight gain (*p* = 0.007). For other diagnosis, changes in weight trend did not demonstrate statistically significant associations with survival outcomes (Table 4).

Of 566 children who gained weight, 6 (1%) abandoned treatment, whereas of 679 children who did not gain weight, 17 (2.5%) abandoned treatment (*p* = 0.088). Fewer children who improved their NS abandoned treatment (2, 0.8%) compared to those without improved NS (21, 2.1%; *p* = 0.198) (Table 2). Specifically, fewer patients with weight gain abandoned treatment (*n* = 17, 2.5%) compared to those without weight gain (*n* = 6, 1.1%; *p* = 0.008). More patients with weight gain are alive and in remission (*n* = 463, 81.8%) compared to those without weight gain (*n* = 507, 75.7%; *p* = 0.002). Fewer patients with weight gain died of disease (*n* = 78, 13.8%) than those without weight gain (*n* = 125, 18.4%; *p* = 0.039) (Table 2).

## Discussion and conclusion

This study aimed to evaluate the impact of NS on short-term clinical outcomes in children with cancer, focusing on the prevalence and trajectory of undernutrition and over-nutrition, and their association with EFS and OS. Key findings include a high prevalence of undernutrition at diagnosis, significant improvement in NS for a subset of children during treatment and better clinical outcomes (EFS and OS) among children who were WN or ON at diagnosis or follow-up or who gained weight during treatment.

Undernutrition is highly prevalent in the general paediatric population in LMICs, with contributory factors being low socioeconomic status, food insecurity, low educational levels, dietary habits, poor access to healthcare, unhygienic living conditions/sanitation and co-morbid conditions like infections [17, 18]. The interplay between cancer and nutrition is complex, with multiple mechanisms for undernutrition in children with cancer, such as increased metabolic rate, anorexia, inflammation and altered physical activity [3–6]. In LMICs, pre-existing undernutrition is further compounded by delayed cancer diagnosis and advanced presentations of disease [12, 19–24, 29].

In this large cohort, two-thirds of children at diagnosis and half at 6-month follow-up were severely or moderately malnourished. Only a small proportion was OW/obese at diagnosis (3.8%), with a marginal increase at follow-up (5.8%). This is similar to literature from LMICs worldwide [7, 19–24]; reports from upper-income and upper-middle-income countries show a lower proportion of undernutrition and higher over-nutrition in children with cancer [9, 25–28] .

In an apparent paradox, obesity and metabolic syndrome are major concern in survivors of childhood cancer, with OW in 20% and metabolic syndrome in 4.2% [30, 31]. In our cohort, children with all tumour diagnoses had 60%–70% prevalence of undernutrition at diagnosis, with the highest being in aggressive malignancies like lymphoma, AML, bone and brain tumours; this is similar to reported literature [12, 19–24].

While a significant proportion of children continued to be UN at 6-month follow-up, nearly half the cohort gained weight >10% from baseline and 20.4% showed an improvement in NS. Weight loss was most pronounced in children with bone and brain tumours, while children with leukaemia, lymphoma and abdominal solid tumours had the greatest improvement in weight and NS. While there is sparse literature on trends in NS by individual tumour types, it is reported that children with solid tumours tend to improve nutritionally once in remission [27]. Children with hematological malignancies on treatment have been reported to show rapid increases in fat mass, with an acute loss of skeletal muscle mass; but in other reports, they were found to be at high risk of developing undernutrition [22, 26]. Children with brain/central nervous system tumours present with symptoms like nausea and vomiting and are known to have significant nutritional morbidity at diagnosis, which worsens during treatment, including cranial radiation [32]. Chemotherapy regimens used in bone tumours tend to be cyclical and invariably cause TRTs like febrile neutropenia, mucositis and other gastro-intestinal disturbances; this is true for several other childhood tumour types as well. These toxicities need careful assessment and optimal nutritional management (including supplements) and medical supportive care to maintain weight [3–6].

In our cohort, the short-term survival outcomes, i.e., EFS at 24 months was higher for children who were WN or ON at diagnosis or follow-upon gained weight during treatment. While the proportion of patients who expired, including due to TRT, was higher in the UN group (at diagnosis and follow-up) and those did not improve their NS, this was not statistically significant. Malnutrition (both under- and over-nutrition) are known to cause inferior outcomes across tumour groups, leading to increased treatment abandonment, causing and worsening TRT (including toxic deaths), increased susceptibility to infections, prolonged hospitalisation, treatment delays and poorer quality of life [3–7, 32–35].

Studies across Central America, South Africa, Malawi and Nicaragua consistently show that undernutrition and food insecurity at diagnosis lead to increased treatment abandonment, higher treatment-related morbidity and significantly inferior EFS and OS. Research from Brazil and other regions suggests that proactive nutritional management can mitigate these negative effects, potentially resulting in stable OS despite pre-treatment undernutrition [19, 20, 22, 23, 25]. Studies from other parts of Africa and India have demonstrated higher mortality due to infections and TRT in UN children [21, 32–34].

Severe malnutrition in children is known to cause multiple organ dysfunctions and varying degrees of immune dysregulation, leading to an increased risk of morbidity and mortality, mainly due to infections [18]. Altered states of nutrition may also affect drug pharmacokinetics and could potentially either increase drug toxicities or reduce treatment efficacy [35]. Other reasons for decreased non-toxic mortality are as yet unclear. Malnutrition may also influence long-term effects of chemotherapy, such as cardiotoxicity and second cancers; cancer treatment may, in turn lead to alterations in body composition, which can affect long-term survival post cancer [3, 5, 34].

There is a growing body of evidence to suggest that remediation of undernutrition can reverse many of these adverse clinical outcomes and result in lower treatment abandonment, lower TRTs, reduce treatment delays and improve EFS [7–9]. This necessitates careful and periodic clinical and nutritional assessment of children with cancer, with arm anthropometry (preferably local standards) being a useful indicator of clinically relevant undernutrition, especially in children with solid tumours [12, 19–21, 36, 37]. Recent literature also focuses on the importance of micronutrient deficiency and altered body composition in children with cancer, and these should be assessed where possible [3]. In our cohort, children improved their NS either because of enteral nutrition support or oral dietary intervention with formula feeds and counseling. Proactive nutritional strategies tailored to the child and using highly nutritious, affordable and home-based nutritional supplementation are proven to be effective in improving NS as well as clinical outcomes [10, 15, 38].

We acknowledge the limitations of this study in that there was no risk-stratification based on disease presentations, weight loss before diagnosis or intensity of treatment received, which would have helped define children at the highest nutritional risk. Although the nutritional and clinical details were recorded prospectively, several nutritionists were involved in taking anthropometric measurements over this period. Importantly, the clinical impact of micronutrient status was not studied.

This study highlights a high baseline prevalence of undernutrition among Indian children with cancer and demonstrates that persistent malnutrition remains a challenge. We observed that patients who gained weight showed trends toward better survival, emphasising the prognostic importance of early and continuous nutritional support.

Moreover, this study reports one of the largest real world datasets from a high-volume center in an LMIC. By providing a broad view of NS and its correlation with survival across various paediatric malignancies, this work serves as a framework for future prospective, risk-stratified studies to identify children at the highest nutritional risk and to evaluate the impact of targeted nutritional interventions on both short- and long-term outcomes.

## List of abbreviations

ALL, Acute lymphoblastic leukaemia; AML, Acute myeloid leukaemia; BMI, Body mass index-for-age; EFS, Event free survival; IQR, Interquartile range; LIC, Low income countries; LMIC, Low middle income countries; MAM, Moderate acute malnutrition; MUAC, Mid upper arm circumference; NS, Nutritional status; ON, Over-nourished; OS, Overall survival; OW, Overweight; SAM, Severe acute malnutrition; SD, Standard deviations; TRT, Treatment-related toxicities; WHO, World Health Organiszation; WN, Well nourished; UN, Undernourished.

## Ethical approval

This study has been presented in part at the Annual Congress of the International Society of Paediatric Oncology (SIOP), Barcelona in October 2022. IEC: Project no 900470 approved 03.02.2021.

## Conflicts of interest

The authors do not report any conflicts of interest.

## Funding

None.

## Author contributions

All authors contributed to the study design, data collection and interpretation, patient management, writing and review of the paper and approval of the final version.

## Data availability

Data will be shared upon request.

## Figures and Tables

**Supplementary Table 1.** NS classification: criteria for defining SAM, MAM, ON and WN status based on WHO and Frisancho standards for children.

Category	Age ≤ 5 Years (WHO)	Age > 5 Years (WHO / Frisancho)
SAM	MUAC < 11.5 cm OR weight-for-height < −3 SD	BMI-for-age < −3 SD OR MUAC < 5th percentile
MAM	MUAC 11.5–12.5 cm OR weight-for-height −3 SD to −2 SD	BMI-for-age −2 SD to −3 SD OR MUAC 5th–10th percentile
ON	Weight-for-height > 1 SD (OW) to > 2 SD (Obese)	BMI-for-age > 1 SD OR MUAC > 50th percentile
WN	All other children meeting standard growth parameters	All other children meeting standard growth parameters

**Supplementary Table 2.** Two-year EFS by diagnosis and NS.

	NS at baseline	2 years EFS	p at baseline	NS at follow-up	2 years EFS	p at follow-up
ALL	WN 178 (69.5%)	74.1% ± 2.80%	0.285	WN 175 (74.2%)	82.5% ± 2.50%	0.941
	UN 355 (64.8%)	73.9% ± 1.90%		UN 189 (73.8%)	81.8% ± 2.50%	
	ON 22 (75.9%)	75.9% ± 7.90%		ON 25 (71.4%)	79.8% ± 6.80%	
AML	WN 21 (65.6%)	65.3% ± 8.50%	0.405	WN 25 (69.4%)	72% ± 7.50%	0.636
	UN 50 (52.1%)	54.6% ± 5.10%		UN 28 (65.1%)	69.2% ± 7.10%	
	ON 2 (66.7%)	50% ± 35.40%		ON 2 (100%)	100%	
Lymphoma	WN 56 (87.5%)	88.9% ± 3.90%	0.084	WN 53 (89.8%)	93.1% ± 3.30%	0.895
	UN 130 (75.6%)	78.9% ± 3.10%		UN 58 (87.9%)	90.7% ± 3.60%	
	ON 13 (92.9%)	91.7% ± 8.00%		ON 18 (90%)	90% ± 6.70%	
Brain tumor	WN 38 (73.1%)	82.2% ± 5.40%	0.095	WN 20 (62.5%)	74.9% ± 77%	0.485
	UN 54 (56.8%)	62.6% ± 5.10%		UN 38 (66.7%)	71.3% ± 6.10%	
	ON 2 (66.7%)	66.7% ± 27.20%		ON 3 (100%)	100%	
Bone tumors	WN 32 (52.5%)	57.8% ± 6.50%	0.289	WN 30 (71.4%)	82.7% ± 6.00%	0.775
	UN 97 (61.8%)	67.7% ± 3.80%		UN 60 (67.4%)	74.2% ± 4.80%	
	ON 8 (72.7%)	71.6% ± 14.00%		ON 2 (66.7%)	66.7% ± 27.20%	
Head-neck tumors	WN 21 (72.4%)	74.8% ± 8.30%	0.458	WN 12 (92.3%)	91.7% ± 8.00%	0.229
	UN 28 (58.3%)	66.6% ± 7.10%		UN 16 (69.6%)	78.3% ± 8.6%	
	ON 3 (50%)	50% ± 20.40%		ON 0	-	
Abdominal tumors	WN 56 (62.2%)	63.7% ± 5.10%	0.373	WN 42 (66.7%)	71.1% ± 5.80%	0.219
	UN 84 (52.2%)	53.8% ± 4.00%		UN 48 (63.2%)	63.6% ± 5.60%	
	ON 6 (54.5%)	51.9% ± 15.7%		ON 6 (100%)	100%	
Other solid tumors	WN 34 (64.2%)￼	61.7% ± 7.00%	0.747	WN 19 (70.4%)	76% ± 8.60%	0.926
	UN 41 (53.2%)	65.8% ± 5.50%		UN 31 (66%)	76.5% ± 6.20%	
	ON 2 (66.7%)	66.7% ± 27.20%		ON 2 (66.7%)	66.7% ± 27.20%	

**Supplementary Table 3.** Two-year OS by diagnosis and NS.

Diagnosis	NS at baseline	2 years OS	*p* at baseline	NS at follow-up	2 years OS	*p* at follow-up
ALL	WN + ON 216 (75.8%)	79.2% ± 2.5%	*p* = 0.147	WN + ON 218 (80.4%	86.3% ± 2.1%	*p* = 0.317
	UN 388 (70.8%)	77.1% ± 1.8%		UN 198 (77.3%)	83.7% ± 2.4%	
AML	WN + ON 25 (71.4%)	68.9% ± 8.2%	*p* = 0.150	WN + ON 29 (76.3%)	76.1% ± 7.0%	*p* = 0.544
	UN 55 (57.3%)	59.3% ± 5.1%		UN 30 (69.8%)	75.7% ± 6.7%	
Abdominal tumor	WN + ON 75 (72.8%)	74.1% ± 4.5%	*p* = 0.107	WN + ON 56 (81.2%)	86.5% ± 4.2%	*p* = 0.184
	UN 100 (62.1%)	63.8% ± 3.9%		UN 56 (73.7%)	73.9% ± 5.2%	
Lymphoma	WN + ON 70 (89.7%)	90.8% ± 3.3%	*p* = 0.114	WN + ON 73 (92.4%)	93.6% ± 2.8%	*p* = 0.744
	UN 140 (81.4%)	82.3% ± 2.9%		UN 62 (93.9%)	93.8% ± 3.0%	
Bone tumor	WN + ON 47 (65.3%)	67.3% ± 5.8%	*p* = 0.538	WN + ON 36 (80%)	88.4% ± 4.9%	*p* = 0.527
	UN 108 (68.8%)	72.9% ± 3.7%		UN 67 (75.3%)	81.1% ± 4.3%	
Brain tumor	WN + ON 43 (75.4%)	81.9% ± 5.2%	*p* = 0.148	WN + ON 29 (80.6%)	83.1% ± 6.3%	*p* = 0.525
	UN 64 (66%)	69.1% ± 4.9%		UN 44 (74.6%)	79.9% ± 5.4%	
Other tumors	WN + ON 40 (70.2%)	73% ± 6.2%	*p* = 0.675	WN + ON 24 (77.4%)	73% ± 6.2%	*p* = 0.464
	UN 56 (70.9%)	77.2% ± 4.9%		UN 39 (79.6%)	77.2% ± 4.9%	
Head-neck tumors	WN + ON 24 (68.6%)	70.2% ± 7.9%	*p* = 0.285	WN + ON 13 (100%)	100%	*p* = 0.188
	UN 39 (81.3%)	80.7% ± 6.2%		UN 20 (87%)	86.5% ± 7.2%	

**Supplementary Table 4.** Institutional protocols of treatment by diagnosis.

Diagnosis	Treatment protocols	References
ALL	ICiCLe protocol (Indian Collaborative Childhood Leukaemia group)	Gogoi MP, Das P, Das N, Das S, Narula G, Trehan A, Bakhshi S, Radhakrishnan V, Seth R, Tembhare P, Singh Sachdeva MU, Chopra A, Sundersingh S, Parihar M, Bhattacharya R, Banavali S, Saha V, Krishnan S. Risk stratified treatment for childhood acute lymphoblastic leukaemia: a multicentre observational study from India. Lancet Reg Health Southeast Asia. 2025 May 13;37:100593. doi: 10.1016/j.lansea.2025.100593. PMID: 40487914
AML	3 + 7 (cytarabine and daunorubicin for 7 days with 3 days of anthracycline) and 3 HiDAC (high-dose cytarabine)	Srinivasan S, Dhamne C, Patkar N, Chatterjee G, Moulik NR, Chichra A, Pallath A, Tembhare P, Shetty D, Subramanian PG, Narula G, Banavali S. KIT exon 17 mutations are predictive of inferior outcome in pediatric acute myeloid leukemia with RUNX1::RUNX1T1. Pediatr Blood Cancer. 2024 Feb;71(2):e30791. doi: 10.1002/pbc.30791. Epub 2023 Nov 28. PMID: 38014874
Hodgkin lymphoma	OEPA-COPDAC chemotherapy (OEPA: vincristine, etoposide, prednisone, and doxorubicin; followed by COPDAC: cyclophosphamide, vincristine, prednisone and dacarbazine) OR ABVD (adriamycin, bleomycin, vinblastine and dacarbazine)	Parambil BC, Narula G, Prasad M, Shah S, Shet T, Shridhar E, Khanna N, Laskar S, Gujral S, Sankaran H, Banavali S. Clinical profile and outcome of classical Hodgkin lymphoma treated with a risk-adapted approach in a tertiary cancer center in India. Pediatr Blood Cancer. 2020 Feb;67(2):e28058. doi: 10.1002/pbc.28058. Epub 2019 Nov 13. PMID: 31724304
Non-hodgkin lymphoma	Modified MCP-842 protocol	Srinivasan S, Roy Moulik N, Kc A, Narula G, Sankaran H, Prasad M, Dhamne C, Cheriyalinkal Parambil B, Shah S, Shet T, Sridhar E, Gujral S, Banavali S. Increased toxicities in children with Burkitt lymphoma treated with rituximab: Experience from a tertiary cancer center in India. Pediatr Blood Cancer. 2020 Nov;67(11):e28682. doi: 10.1002/pbc.28682. Epub 2020 Aug 31. PMID: 32865865
Bone tumours	Ewing Sarcoma: Ewings Family of Tumours (EFT-2001) protocol Osteosarcoma -OGS 2012 protocol	Parambil BC, Khemani P, Puri A, Gulia A, Prasad M, Gollamudi VRM, Ramadwar M, Rekhi B, Panjwani P, Nayak P, Pruthi M, Qureshi S, Purandare N, Janu A, Pawar A, Adhav K, Chinnaswamy G. Outcomes Based on Histological Tumor Necrosis and Predictive Clinical and Laboratory Parameters for Necrosis in Children With Osteosarcoma Treated on a Non-High Dose Methotrexate-Based Chemotherapy Backbone. Pediatr Blood Cancer. 2025 Mar;72(3):e31471. doi: 10.1002/pbc.31471. Epub 2024 Dec 15. PMID: 39676122
Germ cell tumours	Standard PEB (cisplatin, etoposide, bleomycin) chemotherapy	Ramanathan S, Prasad M, Vora T, Parambil BC, Kembhavi S, Ramadwar M, Khanna N, Laskar S, Kurkure P, Qureshi S, Banavali S, Chinnaswamy G. Outcomes and prognostic variables of extracranial germ cell tumors in children and adolescents treated over a decade: A developing world perspective. Pediatr Blood Cancer. 2022 Jul;69(7):e29765. doi: 10.1002/pbc.29765. Epub 2022 May 13. PMID: 35561025
Wilms tumour	Either COG (Children’s Oncology Group) or SIOP (International Society of Pediatric Oncology) protocols	Qureshi SS, Kembhavi SA, Bhagat M, Kapadia T, Prasad M, Vora T, Chinnaswamy G, Ramadwar M, Laskar S, Khanna N, Baheti A, Shah S, Kurkure P, Talole S. Customized approach for upfront or delayed resection using radiological criteria in unilateral, nonmetastatic pediatric renal tumors: A prospective study. Pediatr Blood Cancer. 2019 Aug;66 Suppl 3:e27815. doi: 10.1002/pbc.27815. Epub 2019 May 16. PMID: 31099132
Hepatoblastoma	SIOPEL protocols (Société Internationale d’Oncologie Pédiatrique-Epithelial Liver tumors	Srinivasan S, Prasad M, Parambil BC, Shrimal A, Gollamudi VRM, Subramani V, Ramadwar M, Khanna N, Baheti AD, Gala K, Patil V, Laskar S, Qureshi S, Chinnaswamy G. Treatment outcomes and prognostic factors in children with hepatoblastoma using a risk-stratified approach. Pediatr Blood Cancer. 2023 Jul;70(7):e30302. doi: 10.1002/pbc.30302. Epub 2023 Apr 12. PMID: 37046413
Nasopharyngeal carcinoma	BMP protocol (bleomycin, methotrexate and cisplatin)	Laskar S, Bahl G, Muckaden M, Pai SK, Gupta T, Banavali S, Arora B, Sharma D, Kurkure PA, Ramadwar M, Viswanathan S, Rangarajan V, Qureshi S, Deshpande DD, Shrivastava SK, Dinshaw KA. Nasopharyngeal carcinoma in children: comparison of conventional and intensity-modulated radiotherapy. Int J Radiat Oncol Biol Phys. 2008 Nov 1;72(3):728-36. doi: 10.1016/j.ijrobp.2008.01.032. Epub 2008 Apr 18. PMID: 18374512
Rhabdomyosarcoma	IRS-IV protocol (Intergroup Rhabdomyosarcoma Study-IV)	Ramanathan S, Sisodiya S, Shetty O, Prasad M, Parambil BC, Shah S, Ramadwar M, Khanna N, Laskar S, Qureshi S, Vora T, Chinnaswamy G. Outcome and prognostic variables in childhood rhabdomyosarcoma (RMS) with emphasis on impact of FOXO1 fusions in non-metastatic RMS: experience from a tertiary cancer centre in India. Ecancermedicalscience. 2023 Apr 27;17:1539. doi: 10.3332/ecancer.2023.1539. PMID: 37138963; PMCID: PMC10151086
Retinoblastoma	VEC = 3 weekly cycles of vincristine, etoposide and cyclophosphamide	
Soft tissue sarcomas	IVAdo - 3 weekly cycles of ifosfamide, vincristine and doxorubicin	
Brain tumours	Medullblastoma: CET (cyclophosphamide, cisplatin, etoposide); CEJ protocol for younger children; LGG - VCR+Carboplatin OR single agent Vinblastin; Ependymoma: CEJ protocol for younger children	Gupta T, Sinha S, Chinnaswamy G, Vora T, Prasad M, Bhat V, Goda JS, Krishnatry R, Chatterjee A, Epari S, Sahay A, Moiyadi A, Shetty P, Patil V, Khatua S, Jalali R, Kurkure P. Safety and efficacy of concurrent carboplatin during full-dose craniospinal irradiation for high-risk/metastatic medulloblastoma in a resource-limited setting. Pediatr Blood Cancer. 2021 May;68(5):e28925. doi: 10.1002/pbc.28925. Epub 2021 Feb 3. PMID: 33533557
Langerhans cell histiocytosis (LCH)	In-house vinblastine and steroids-based protocol	Narula G, Pradhan ND, Arora B, Banavali SD. Treatment of Langerhans cell histiocytosis with a modified risk-adapted protocol-experience from a tertiary cancer institute in India. Pediatr Blood Cancer. 2018 Aug;65(8):e27028. doi: 10.1002/pbc.27028. Epub 2018 Mar 7. PMID: 29512864

**Table 1.** Demographics, diagnoses and outcomes of the cohort (n = 2,083).

Demographics	Prevalence
Median age (IQR)	6.5 years (3.3–11 years)
Male: Female ratio	2.2:1
Diagnoses	*n* = 2,083
ALL	834 (40%)
Abdominal tumors	265 (12.7%)
Lymphoma	250 (12%)
Bone tumor	230 (11%)
Brain tumor	154 (7.4%)
Other tumors	136 (6.6%)
AML	131 (6.3%)
Head-neck tumors	83 (4%)
Outcome	*n* = 2,083
Alive, in remission	1,322 (63.5%)*
Alive, Relapsed	106 (5.1%)
Expired	590 (28.3%)
Expired due to disease progression	412
Expired due to toxic death	151
Abandoned treatment	65 (3.1%)

*includes both first and second remission

**Table 2.** Clinical outcomes of the entire cohort by NS at diagnosis, follow-up and weight changes during treatment.

	NS	N	Alive, in remission (either first or second remission)		Expired			Abandoned Treatment	
					Disease death	Toxic death	p		
NS at diagnosis	WN	643	459 (71.4%)	*p* = 0.404	125 (17.3%)*	57 (7.9%)*	*p* = 0.61	22 (3.4%)	*p* = 0.404
	ON	80	58 (72.5%)					2 (2.5%)	
	UN	1360	911 (67%)		287 (21.1%)	121 (8.9%)		41 (3%)	
NS at follow-up	WN	511	407 (79.6%)	*p* = 0.542	84 (18.1%)*	20 (3.4%)*	*p* = 0.187	8 (1.6%)	*p* = 0.542
	ON	72	60 (83.3%)					2 (2.8%)	
	UN	662	501 (75.7%)		120 (22.9%)	25 (4.8%)		13 (2%)	
Weight gain (*n* = 1,245)	Yes	566	463 (81.8%)	*p* = 0.002	78 (13.8%)	17 (3%)	*p* = 0.039	6 (1.1%)	*p* = 0.088
	No	679	507 (74.8%)		125 (18.4%)	28 (4.1%)		17 (2.5%)	
Weight loss (*n* = 1,245)	Yes	145	115 (79.3%)	*p* = 0.188	28 (19.3%)	2 (1.4%)	*p* = 0.204	0	*p* = 0.098
	No	1100	854 (77.8%)		175 (15.9%)	43 (3.9%)		23 (2.1%)	
Improved NS (*n* = 1,245)	Yes	254	208 (81.9%)	*p* = 0.09	37 (14.6%)	6 (2.4%)	*p* = 0.306	2 (0.8%)	*p* = 0.198
	No	991	761 (76.9%)		166 (16.8%)	39 (3.9%)		21 (2.1%)	

WN =well-nourished (includes WN and mildly malnourished); UN = undernourished (includes moderately malnourished and severely malnourished); ON=overnourished (includes OW and obese)*Owing to small numbers, with WN status and ON status have been clubbed together in selected analyses

**Table 3.** Nutritional status at diagnosis and follow-up.

Diagnosis	Number at diagnosis	NS at diagnosis (n = 2,083)			NS at 3-month follow-up (n = 1,176)			NS at 6-month follow-up (n = 1,006)		
		**WN**	**UN**	**ON**	**WN**	**UN**	**ON**	**WN**	**UN**	**ON**
All patients	2,083	643 (30.9%)	1,360 (65.3%)	80 (3.8%)	433 (36.8%)	698 (59.4%)	45 (3.8%)	419 (41.7%)	525 (52.2%)	62 (6.1%)
ALL	834	256 (30.7%)	549 (65.8%)	29 (3.5%)	194 (37.7%)	297 (57.7%)	24 (4.6%)	196 (45.3%)	206 (47.6%)	31 (7.1%)
AML	131	32 (24.4%)	96 (73.3%)	3 (2.3%)	33 (45.2%)	40 (54.8%)	0	29 (45.3%)	33 (51.6%)	2 (3.1%)
Lymphoma*	250	64 (25.6%)	172 (68.8%)	14 (5.6%)	48 (35.5%)	80 (59.3%)	7 (5.2%)	52 (47.7%)	40 (36.7%)	17 (15.6%)
Brain tumor	154	54 (35.1%)	97 (63%)	3 (1.9%)	27 (31.8%)	57 (67.1%)	1 (1.2%)	28 (34.6%)	50 (61.7%)	3 (3.7%)
Head-neck tumors**	83	29 (34.9%)	48 (57.9%)	6 (7.2%)	13 (38.2%)	21 (61.8%)	0	9 (31%)	20 (69%)	0
Bone tumor	230	61 (26.5%)	158 (68.7%)	11 (4.8%)	38 (30.2%)	82 (65.1%)	6 (4.7%)	33 (30.3%)	75 (68.8%)	1 (0.9%)
Abdominal tumors	265	93 (35%)	161 (60.8%)	11 (4.2%)	51 (38.6%)	77 (58.4%)	4 (3%)	48 (42.9%)	59 (52.7%)	5 (4.4%)
Other solid tumors	136	54 (39.7%)	79 (58.1%)	3 (2.2%)	29 (38.2%)	44 (57.9%)	3 (3.9%)	24 (34.8%)	42 (60.9%)	3 (4.3%)

WN =well-nourished (includes WN and mildly malnourished); UN = undernourished (includes moderately malnourished and severely malnourished); ON=overnourished (includes OW and obese)*Includes Hodgkin and Non-Hodgkin lymphoma **non-central nervous system head-neck tumours

**Table 4.** Survival as per trends in weight.

Diagnosis	Weight trend	n	2 years OS	p	n	2 years EFS	p
ALL	Weight gain	201 (82.4%)	88.5% ± 2.10%	*p* = 0.045	185 (75.8%)	85.2% ± 2.30%	*p* = 0.145
	No weight gain	218 (76.2%)	82.1% ± 2.30%		206 (72%)	78.8% ± 2.50%	
	Weight loss	42 (73.7)	83.6% ± 5.00%	*p* = 0.373	40 (70.2%)	78.2% ± 5.60%	*p* = 0.519
	No weight loss	378 (79.7%)	85.3% ± 1.70%		352 (74.3%)	82.3% ± 1.80%	
	Improved NS	92 (84.4%)	91.5% ± 2.70%	*p* = 0.064	84 (77.1%)	87.8% ± 3.20%	*p* = 0.172
	No improved NS	327 (77.7%)	83.3% ± 1.90%		307 (72.9%)	80.2% ± 2.00%	
AML	Weight gain	25 (71.4%)	77% ± 7.10%	*p* = 0.955	24 (68.6%)	74.2% ± 7.40%	*p* = 0.828
	No weight gain	34 (73.9%)	75.1% ± 6.50%		31 (67.4%)	68.9% ± 6.90%	
	Weight loss	4 (80%)	66.7% ± 27.20%	*p* = 0.855	4 (80%)	66.7% ± 27.20%	*p* = 0.677
	No weight loss	55 (72.4%)	76% ± 4.90%		51 (67.1%)	70.9% ± 5.20%	
	Improved NS	11 (64.7%)	63.5% ± 12.00%	*p* = 0.379	10 (58.8%)	64.2% ± 11.80%	*p* = 0.353
	No improved NS	48 (75%)	79.2% ± 5.10%		45 (70.3%)	73.1% ± 5.60%	
Abdominal tumor	Weight gain	55 (78.6%)	82.5% ± 4.60%	*p* = 0.471	48 (68.6%)	71% ± 5.50%	*p* = 0.385
	No weight gain	56 (75.7%)	77.2% ± 5.00%		47 (63.5%)	65.5% ± 5.60%	
	Weight loss	13 (92.9%)	92.3% ± 7.40%	*p* = 0.186	12 (85.7%)	92.3% ± 7.40%	*p* = 0.144
	No weight loss	98 (75.4%)	78.5% ± 3.70%		83 (63.8%)	65.7% ± 4.20%	
	Improved NS	24 (75%)	81.1% ± 6.90%	*p* = 0.955	20 (62.5%)	65.6% ± 8.40%	*p* = 0.699
	No improved NS	87 (77.7%)	79.4% ± 3.90%		75 (67%)	68.9% ± 4.40%	
Lymphoma	Weight gain	82 (94.3%)	95.4% ± 2.30%	*p* = 0.435	77 (88.5%)	93.1% ± 2.70%	*p* = 0.963
	No weight gain	53 (91.4%)	90.9% ± 3.90%		52 (89.7%)	89.3% ± 4.10%	
	Weight loss	12 (92.3%)	91.7% ± 8.00%	*p* = 0.924	12 (92.3%)	91.7% ± 8.00%	*p* = 0.695
	No weight loss	123 (93.2%)	93.8% ± 2.10%		117 (88.6%)	91.6% ± 2.40%	
	Improved NS	44 (93.6%)	93.6% ± 3.60%	*p* = 0.812	43 (91.5%)	93.6% ± 3.60%	*p* = 0.421
	No improved NS	91 (92.9%)	93.7% ± 2.50%		86 (87.8%)	90.7% ± 3.00%	
Bone tumor	Weight gain: yes	35 (79.5%)	88.6% ± 4.80%	*p* = 0.471	31 (70.5%)	81.7% ± 5.90%	*p* = 0.618
	No weight gain	68 (76.4%)	80.7% ± 4.40%		61 (68.5%)	73.9% ± 4.80%	
	Weight loss yes	19 (76%)	74.2% ± 9.10%	*p* = 0.723	17 (68%)	69.2% ± 9.70%	*p* = 0.795
	No weight loss	82 (77.4%)	85.3% ± 3.50%		73 (68.9%)	77.7% ± 4.10%	
	Improved NS	18 (85.7%)	95.2% ± 4.60%	*p* = 0.257	17 (81%)	89.9% ± 6.80%	*p* = 0.138
	No improved NS	84 (75.7%)	81% ± 3.80%		74 (66.7%)	73.9% ± 4.30%	
Brain tumor	Weight gain	30 (76.9%)	81.5% ± 6.30%	*p* = 0.819	25 (65.8%)	76.1% ± 7.00%	*p* = 0.794
	No weight gain	43 (76.8%)	80.9% ± 5.40%		36 (66.7%)	71.8% ± 6.20%	
	Weight loss	15 (75%)	78.9% ± 9.40%	*p* = 0.822	13 (65%)	70% ± 10.20%	*p* = 0.681
	No weight loss	58 (77.3%)	81.8% ± 4.60%		48 (66.7%)	74.5% ± 5.20%	
	Improved NS	8 (66.7%)	75% ± 12.50%	*p* = 0.429	5 (41.7%)	66.7% ± 13.60%	*p* = 0.069
	No improved NS	65 (78.3%)	82.1% ± 4.40%		56 (70%)	74.6% ± 4.90%	
Othe￼r tumors	Weight gain	32 (91.4%)	91.3% ± 4.80%	p = 0.007	26 (76.5%)	85.3% ± 6.10%	p = 0.094
	No weight gain	31 (68.9%)	81.1% ± 6.00%		26 (60.5%)	68.9% ± 7.20%	
	Weight loss	7 (100%)	100%	*p* = 0.251	7 (100%)	100%	*p* = 0.125
	No weight loss	56 (76.7%)	84.3% ± 4.40%		45 (64.3%)	73.8% ± 5.30%	
	Improved NS	10 (83.3%)	81.8% ± 11.60%	*p* = 0.610	7 (58.3%)	81.8% ± 11.60%	*p* = 0.564
	No improved NS	53 (77.9%)	86.2% ± 4.50%		45 (69.2%)	75% ± 5.40%	
Head-neck tumors	Weight gain	11 (91.7%)	90.9% ± 8.70%	*p* = 0.990	9 (75%)	75% ± 12.50%	*p* = 0.694
	No weight gain	22 (91.7%)	91.5% ± 5.80%		19 (79.2%)	87.1% ± 6.90%	
	Weight loss	3 (75%)	75% ± 21.70%	*p* = 0.172	2 (50%)	75% ± 21.70%	*p* = 0.076
	No weight loss	30 (93.8%)	93.3% ± 4.60%		26 (81.3%)	87.1% ± 6.00%	
	Improved NS	4 (100%)	100%	*p* = 0.524	3 (75%)	75% ± 21.70%	*p* = 0.904
	No improved NS	29 (90.6%)	90.2% ± 5.40%		25 (78.1%)	84% ± 6.60%	
